# Standardized pre-bronchoscopy mechanical power and KPC resistance as predictors of mortality in ventilated ICU patients: a retrospective cohort study

**DOI:** 10.3389/fmed.2025.1706376

**Published:** 2026-02-12

**Authors:** Killen H. Briones-Claudett, Killen H. Briones-Zamora, Mónica H. Briones-Claudett, María A. Touriz Bonifaz, Anahí D. Briones-Zamora, Diana C. Briones Marquez, Michelle Grunauer

**Affiliations:** 1 Facultad de Ciencias de la Salud, Universidad Internacional del Ecuador. UIDE, Quito, Ecuador; 2 Briones PulmoCare, Guayaquil, Ecuador; 3 Universidad de Especialidades Espíritu Santo, Samborondón, Ecuador; 4 Intensive Care Unit, Ecuadorian Institute of Social Security (IESS), Babahoyo, Ecuador; 5 Facultad de Ciencias de la Salud, Universidad Católica Santiago de Guayaquil, Guayaquil, Ecuador; 6 Facultad de Ciencias Médicas, Universidad de Guayaquil, Guayaquil, Ecuador; 7 OMNI Hospital, Guayaquil, Ecuador; 8 School of Medicine, Universidad San Francisco de Quito, Quito, Ecuador

**Keywords:** mechanical power, fiberoptic bronchoscopy, mechanical ventilation, intensive care unit (ICU), KPC, mortality, respiratory mechanics

## Abstract

**Background:**

Mechanical power (MP) quantifies the energy delivered by the ventilator per unit time and is linked to ventilator-induced lung injury and mortality in mechanically ventilated patients. Its prognostic value before high-risk interventions such as fiberoptic bronchoscopy remains uncertain. Standardization of pre-procedural ventilator settings may enhance the reliability of respiratory mechanics and MP assessment.

**Methods:**

We conducted a retrospective, single-center cohort study of 30 ICU patients on invasive mechanical ventilation undergoing urgent bronchoscopy. A five-minute stabilization under volume-controlled ventilation with FiO₂ 1.0 and unchanged PEEP and respiratory rate was applied before scope insertion. Pre-procedure MP, gas exchange, and respiratory mechanics were recorded. The primary outcome was 28-day mortality. Logistic regression evaluated associations with clinical and microbiological predictors.

**Results:**

Median age was 66 years and 73% of patients were male. Median baseline MP was 13.6 J/min. Although MP ≥ 18 J/min was associated with impaired respiratory mechanics, it was not linked to mortality. In multivariable analysis, only *Klebsiella pneumoniae* carbapenemase (KPC) positivity independently predicted death (OR 14.6; 95% CI 1.8 − 116.5; *p* = 0.011), whereas MP was non-interpretable. Overall mortality was 26.7%.

**Interpretation:**

In critically ill ventilated patients undergoing urgent bronchoscopy, baseline MP under standardized ventilatory conditions did not independently predict outcome. Instead, KPC positivity emerged as the predominant determinant of mortality. These findings underscore the prognostic dominance of microbiological resistance over transient physiologic parameters and highlight the need to integrate rapid resistance profiling with ventilatory monitoring for risk stratification in ICU bronchoscopy critical need to integrate rapid resistance profiling, such as multiplex PCR for resistance gene detection, alongside standardized ventilatory monitoring for early risk stratification and targeted therapeutic intervention in ICU bronchoscopy.

## Introduction

Fiberoptic bronchoscopy (FOB) is a common diagnostic and therapeutic procedure in the intensive care unit (ICU), enabling airway inspection, secretion clearance, and targeted sampling in patients receiving invasive mechanical ventilation (IMV). Although generally safe, FOB can transiently worsen respiratory mechanics and gas exchange, posing risks in patients with limited pulmonary reserve (1).

Mechanical power (MP) quantifies the energy transferred from the ventilator to the respiratory system per unit time by integrating tidal volume, pressure, respiratory rate, and resistance. Elevated MP is a key driver of ventilator-induced lung injury (VILI) and has been associated with increased mortality in ARDS and broader ICU populations (2). While both magnitude and duration of MP exposure independently predict outcomes (3), most studies have evaluated prolonged time-weighted averages rather than baseline MP immediately prior to high-risk interventions such as FOB.

Bronchoscopic procedures induce marked changes in respiratory mechanics, yet patients usually undergo FOB under individualized ventilator settings. This variability in baseline MP may influence procedural safety and outcomes but has been insufficiently addressed (4). Accurate MP measurement requires a standardized stabilization period immediately before bronchoscopy, using volume-controlled ventilation with fixed FiO₂ and unchanged settings, to minimize confounding by transient manipulations. This approach improves the reliability of MP as a clinical risk marker.

Multidrug-resistant infections—particularly those caused by carbapenemase-producing *Klebsiella pneumoniae* (KPC)—represent a critical determinant of prognosis in mechanically ventilated ICU patients. (5). Recent multicenter reports highlight the importance of rapid detection and targeted therapy to improve survival (6). Molecular diagnostic platforms, including multiplex PCR panels capable of detecting resistance genes within 1–2 h, have emerged as essential tools for early pathogen identification and antimicrobial stewardship (7). The integration of rapid microbiological profiling with standardized physiologic assessment may provide a more comprehensive approach to risk stratification in critically ill patients undergoing invasive procedures.

To address these gaps, we performed a retrospective, single-center cohort study to evaluate whether baseline MP measured under standardized pre-procedural ventilator conditions independently predicts short-term outcomes in ICU patients undergoing urgent bronchoscopy. We hypothesized that higher pre-bronchoscopy MP would be associated with increased mortality and prolonged ventilation, independent of microbiological resistance status.

## Materials and methods

### Study design

We conducted a retrospective, single-center cohort study in a mixed medical–surgical ICU with a prestructured protocol for urgent bronchoscopy and ventilatory management. The study included consecutive cases from January to December 2019.

### Eligibility criteria

Eligible patients were adults (≥18 years) under invasive mechanical ventilation who underwent urgent bedside bronchoscopy with complete ventilatory waveform records immediately prior to the procedure. Patients were excluded if pre-bronchoscopy mechanics data were incomplete or if more than one bronchoscopic episode occurred (only the first analyzed). Patients with refractory instability (dual high-dose vasopressors or PaO₂/FiO₂ < 150 on FiO₂ > 0.80) were excluded from FOB.

### Bronchoscopy indications

FOB was indicated for suspected ventilator-associated pneumonia with new or progressive radiographic infiltrates and purulent secretions, unexplained hemoptysis, suspected endobronchial obstruction, or acute respiratory deterioration with unexplained radiologic abnormalities (8).

### Procedural conduct

Bedside FOB was performed by critical care pulmonologists using a flexible 2.5-mm Pentax^®^ bronchoscope via sealed endotracheal adapter. Sedation (propofol or midazolam), analgesia (fentanyl or remifentanil), and neuromuscular blockade (rocuronium) were used as clinically indicated. Standard monitoring included ECG, invasive blood pressure, pulse oximetry, and ventilator waveform signals (9).

### Ventilatory standardization

A 5-min stabilization interval was applied using volume-controlled ventilation with square-wave flow before scope insertion. During the final 60–90 s, FiO₂ was fixed at 1.0, PEEP and respiratory rate remained unchanged, and no suctioning, instillation, or airway instrumentation was performed. Approximately 30 consecutive ventilatory cycles were exported for analysis (10).

### Respiratory mechanics and mechanical power

Measured variables included driving pressure (ΔP = Pplat − PEEP), static compliance (C_RS = VT/ΔP), elastance (E_RS = 1/C_RS), airway resistance (R_aw), and ventilatory ratio (11).

Mechanical power (MP) was calculated for volume-controlled ventilation using:


MP=0.098×VT(L)×RR×[Ppeak−ΔP/2]

Where VT = exhaled tidal volume (L), RR = rate (breaths·min^−1^), P_peak = peak inspiratory pressure (cmH₂O), ΔP = driving pressure (cmH₂O). MP was expressed in J·min^−1^, categorized into quartiles, with MP ≥ 18 J·min^−1^ (“high”) as per outcome-based evidence (3).

### Bronchoalveolar sampling and microbiology

After stabilization, the bronchoscope advanced for BAL (three 40-mL aliquots, first discarded). BAL underwent standard culture (biochemical/MALDI-TOF ID) (12) and multiplex PCR (FilmArray^®^ Pneumonia Panel) for common respiratory pathogens and resistance genes (7). Pathogens were classified as KPC-positive, other MDR, non-resistant, or negative.

### Outcomes

The primary outcome was 28-day mortality. Secondary outcomes included (1) duration of invasive mechanical ventilation, (2) ICU length of stay, and (3) procedural safety events (hypoxemia, bronchospasm, hypotension).

### Statistical analysis

Data distributions were assessed using the Shapiro–Wilk test. Continuous variables were summarized as median [IQR]; categorical variables as *n* (%). Survivors vs. non-survivors were compared with Mann–Whitney U or Student’s *t*-test for continuous variables, and χ^2^ or Fisher’s exact test for categorical variables. Mechanical power was analyzed as both continuous and dichotomized (≥18 vs. < 18 J/min). Kaplan–Meier survival analysis was performed stratified by MP, with log-rank test for group comparisons (13). Logistic regression (univariable and multivariable) tested associations of MP and microbiological resistance. Multivariable models were limited to two predictors, consistent with the ≥10 events per variable rule.

## Results

**Figure 1:**
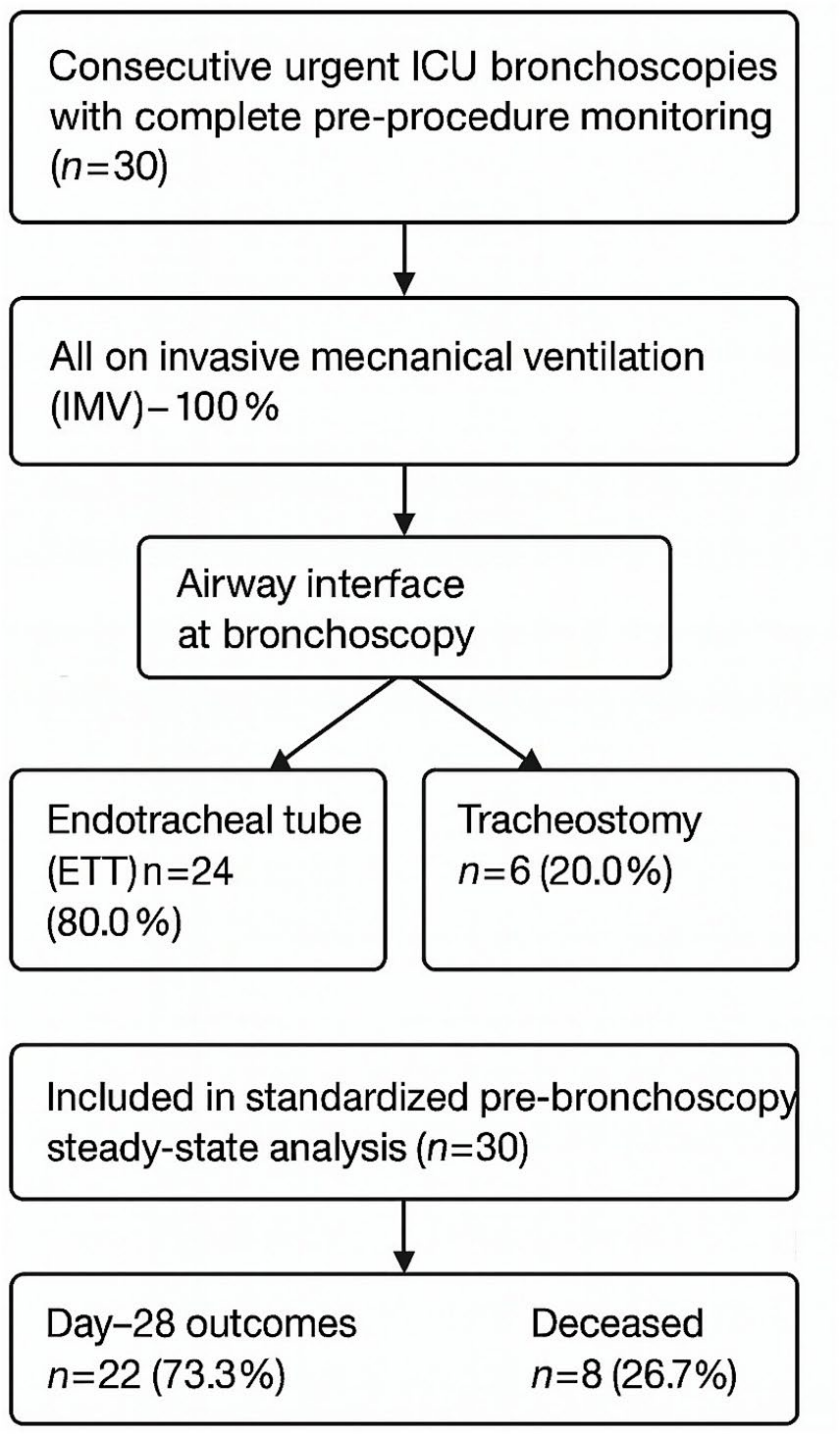
Study flow diagram. Flow of patient inclusion and exclusion criteria for urgent fiberoptic bronchoscopy. Final analytic cohort consisted of 30 mechanically ventilated patients, of whom 22 survived and 8 died by day 28.

A total of 30 urgent fiberoptic bronchoscopies performed in invasively ventilated patients were analyzed. At the time of bronchoscopy, 80% of patients were intubated via endotracheal tube and 20% via tracheostomy. By day 28, survival was 73.3%, while 26.7% of patients had died. (Figure 1).

### Baseline characteristics

Demographic, pharmacologic, and procedural characteristics are summarized in Table 1. There were no significant differences between survivors and non-survivors with respect to age, sex, airway interface, sedation and analgesia, neuromuscular blockade, or adjunctive therapies. The incidence of procedural complications was low, with hypoxemia (10%), bronchospasm (40%), and atrial fibrillation (6.7%) occurring infrequently and without survival differences.

**Table 1:** Baseline, procedural, and pharmacologic characteristics of the study cohort.

Variable	All patients (*n* = 30)	Survivors (*n* = 22)	Non-survivors (*n* = 8)	*p*-value
Age, years, median [IQR]	66 [58–72]	65 [57–70]	68 [60–74]	0.41
Male sex, n (%)	22 (73.3)	16 (72.7)	6 (75.0)	0.89
Airway interface, *n* (%)				
ETT	24 (80.0)	18 (81.8)	6 (75.0)	0.68
Tracheostomy	6 (20.0)	4 (18.2)	2 (25.0)	
Ventilatory mode: VCV, n (%)	30 (100)	22 (100)	8 (100)	—
Sedation/analgesia, *n* (%)				
Propofol	20 (66.7)	15 (68.2)	5 (62.5)	0.77
Midazolam	10 (33.3)	7 (31.8)	3 (37.5)	
Fentanyl/remifentanil	28 (93.3)	20 (90.9)	8 (100)	0.42
Neuromuscular blockade (rocuronium)	12 (40.0)	9 (40.9)	3 (37.5)	0.85
Vasopressor use, *n* (%)	14 (46.7)	9 (40.9)	5 (62.5)	0.30
Hydrocortisone use, *n* (%)	8 (26.7)	5 (22.7)	3 (37.5)	0.38
Safety events, *n* (%)				
Hypoxemia	3 (10.0)	2 (9.1)	1 (12.5)	0.78
Bronchospasm	12 (40.0)	9 (40.9)	3 (37.5)	0.87
Atrial fibrillation	2 (6.7)	1 (4.5)	1 (12.5)	0.46

Values are presented as *n* (%) or median [IQR]. Group comparisons were performed using χ^2^ or Fisher’s exact test for categorical variables, and Mann–Whitney U test for continuous variables. ETT, endotracheal tube; VCV, volume-controlled ventilation.

### Gas exchange and respiratory mechanics

During the standardized 5-min pre-bronchoscopy stabilization period, gas exchange parameters and respiratory mechanics showed no significant differences between survivors and non-survivors (Table 2). Median driving pressure, compliance, elastance, airway resistance, and mechanical power were comparable between groups.

**Table 2:** Gas exchange and respiratory mechanics during standardized pre-bronchoscopy ventilation.

Variable	All patients (*n* = 30)	Survivors (*n* = 22)	Non-survivors (*n* = 8)	*p*-value
pH	7.34 [7.28–7.39]	7.35 [7.29–7.40]	7.32 [7.27–7.36]	0.28
PaCO₂, mmHg	46 [40–54]	45 [39–52]	48 [42–57]	0.33
PaO₂, mmHg	82 [70–94]	84 [72–96]	79 [68–89]	0.41
HCO₃⁻, mmol/L	23 [21–26]	24 [22–26]	21 [19–24]	0.09
Base excess, mmol/L	–1 [−3 − +1]	–1 [−2 − +2]	–3 [−5 – –1]	0.08
ΔP (Driving pressure), cmH₂O	13 [11–15]	12 [11–14]	14 [12–16]	0.21
C_RS (Compliance), mL·cmH₂O⁻^1^	35 [30–40]	36 [32–42]	32 [28–36]	0.18
E_RS (Elastance), cmH₂O·L⁻^1^	28 [24–33]	27 [23–31]	31 [27–36]	0.22
R_aw (Airway resistance), cmH₂O/L/s	12 [10–15]	11 [9–14]	13 [11–16]	0.27
Ventilatory ratio	1.6 [1.3–1.9]	1.5 [1.3–1.8]	1.7 [1.4–2.0]	0.19
Tidal volume, mL/kg PBW	6.5 [6.2–6.9]	6.5 [6.1–6.8]	6.6 [6.3–7.0]	0.35
PEEP, cmH₂O	8 [7–10]	8 [7–10]	9 [8–10]	0.29
MP (Mechanical power), J·min⁻^1^	13.6 [11.5–15.8]	13.2 [11.2–15.0]	14.5 [12.0–16.2]	0.23
Energy per breath, J	0.68 [0.55–0.80]	0.66 [0.53–0.78]	0.72 [0.58–0.83]	0.26

Data are expressed as median [IQR]. Comparisons between survivors and non-survivors used Mann–Whitney U test. Abbreviations: PaO₂, arterial partial pressure of oxygen; PaCO₂, arterial partial pressure of carbon dioxide; HCO₃⁻, bicarbonate; ΔP, driving pressure; C_RS, static compliance of respiratory system; E_RS, elastance of respiratory system; R_aw, airway resistance; VR, ventilatory ratio; MP, mechanical power; PEEP, positive end-expiratory pressure; PBW, predicted body weight.

### Mechanical power stratification

**Figure 2:**
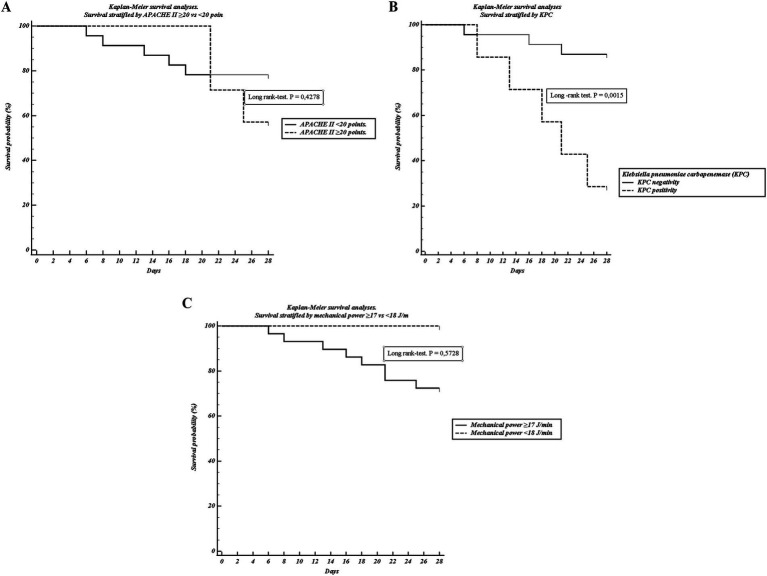
Kaplan–Meier survival analyses. **(A)** Survival stratified by APACHE II ≥ 20 vs. < 20 points. **(B)** Survival stratified by KPC positivity. **(C)** Survival stratified by mechanical power ≥17 vs. < 18 J/min. Curves were compared using the log-rank test. Only KPC positivity was significantly associated with reduced survival.

Stratification of the cohort by mechanical power threshold (≤17 vs. ≥ 18 J/min) revealed that patients with higher MP demonstrated significantly impaired respiratory mechanics, including reduced compliance, elevated driving pressure, and greater elastance (Table 3). Despite these physiological differences, Kaplan–Meier analysis showed no significant separation of survival curves by MP threshold. (Figure 2C).

**Table 3:** Respiratory mechanics, gas exchange, and outcomes stratified by mechanical power (≤17 vs. ≥ 18 J/min).

Variable	Mechanical power ≤18 J/min (*n* = 22)	Mechanical power > 18 J/min (*n* = 8)	*p*-value (Mann–Whitney U)
Compliance (mL·cmH₂O⁻^1^)	40 (38–42)	34.5 (33.6–36.0)	0.0001
Driving pressure ΔP (cmH₂O)	13 (12–13)	15 (13.8–16.0)	0.0001
Elastance E_RS (cmH₂O·L⁻^1^)	26.7 (26.2–27.4)	28.2 (27.3–28.9)	0.0128
Airway resistance R_aw	9.4 (8.4–9.7)	9.7 (9.7–10.4)	0.0114
Energy per breath (J)	3.1 (2.8–3.2)	3.7 (3.5–4.0)	0.0001
Mechanical Power (J/min)	11.9 (10.3–13.4)	19.4 (18.4–20.3)	<0.0001
Ventilatory Ratio (VR)	21.8 (19.8–23.8)	31.4 (29.6–34.2)	<0.0001
PEEP (cmH₂O)	9 (9–10)	12 (12–12.4)	0.0001
Plateau pressure (cmH₂O)	22 (21–23)	26.5 (25.8–27.4)	<0.0001
Exhaled VT (mL)	472 (460–480)	515 (500–526)	0.0001
Minute ventilation (L/min)	8.9 (8.4–9.2)	10.7 (10.1–11.1)	0.0001
pH (baseline)	7.33 (7.29–7.38)	7.34 (7.30–7.41)	0.7245
PaCO₂ (mmHg)	46.1 (42.9–48.3)	48.5 (39.9–50.5)	0.4671
PaO₂ (mmHg)	144 (139–154)	131 (118–152)	0.0515
HCO₃⁻ (mmol/L)	22.2 (19.8–23.6)	22.0 (20.0–25.3)	0.7783
Base excess (mmol/L)	−1.2 (−2.8–0.6)	−1.3 (−3.8–1.4)	0.5024
ICU length of stay, days	14 (10.9–19.1)	11 (7.6–16.1)	0.2589
Duration of mechanical ventilation, days	7 (5.0–8.1)	6.5 (4.8–8.0)	0.6022
APACHE II score	19 (17.9–20.1)	18 (17.8–20.4)	0.9054

Data are expressed as median (IQR). Comparisons between MP ≤17 J/min and MP ≥18 J/min groups were performed using Mann–Whitney U test. ΔP, driving pressure; E_RS, elastance of respiratory system; R_aw, airway resistance; VR, ventilatory ratio; MP, mechanical power; PEEP, positive end-expiratory pressure; VT, tidal volume; PaO₂, arterial partial pressure of oxygen; PaCO₂, arterial partial pressure of carbon dioxide; HCO₃⁻, bicarbonate; ICU, intensive care unit; APACHE II, Acute Physiology and Chronic Health Evaluation II score. Values are presented as median (IQR). Comparisons between groups were made using the Mann–Whitney U test.

### Microbiological and clinical predictors

In bivariate analysis, the presence of KPC-producing *Klebsiella pneumoniae* was strongly associated with 28-day mortality, occurring in 62.5% of non-survivors compared with only 9.1% of survivors (*p* = 0.003). Lower leukocyte counts and higher baseline bicarbonate were also observed among non-survivors (Table 4). By contrast, APACHE II ≥ 20 was not associated with outcome. Kaplan–Meier survival analysis confirmed a pronounced disadvantage in KPC-positive patients (Figure 2B), while APACHE II ≥ 20 showed only a modest and non-significant separation (Figure 2A).

**Table 4:** Bivariate analysis of clinical, microbiological, and physiological variables associated with 28-day mortality.

Variable	Non-survivors (*n* = 8)	Survivors (*n* = 22)	*p*-value
KPC	5 (62.5%)	2 (9.1%)	0.0026
Resistance mechanisms	KPC: 5, No isolate: 3	No resistant: 11; KPC: 1; mecA/lukPV: 4; No isolate: 5; MRSA+KPC: 1	0.0028
Enterococcus	0	2 (9.1%)	0.0170
Pseudomonas	1 (12.5%)	3 (13.6%)	0.0210
HCO₃⁻, mmol/L	23.5 (22.1–26.8)	20.8 (19.8–22.7)	0.0488
APACHE II ≥ 20 points	8 (26.7%)	22 (73.3%)	0,344
Leukocytes (10^3^/μL)	11.3 (8.8–12.8)	13.6 (12.7–14.8)	0.0177
Mechanical Power ≥18 J/min	6/8 (75.0%)	2/22 (9.1%)	<0.001

Data are presented as n (%) or median (IQR). Comparisons between survivors and non-survivors were performed using Fisher’s exact test for categorical variables and Mann–Whitney U test for continuous variables. KPC, *Klebsiella pneumoniae* carbapenemase; MRSA, methicillin-resistant *Staphylococcus aureus*; lukPV, Panton–Valentine leukocidin.

### Multivariable analysis

In the logistic regression model including KPC positivity, APACHE II ≥ 20, and MP ≥ 18 J/min, only KPC positivity remained independently associated with 28-day mortality (OR 14.6, 95% CI 1.8 − 116.5; *p* = 0.011) (Table 5). Neither APACHE II ≥ 20 (OR 1.50, 95% CI 0.17–13.6; *p* = 0.717) nor high MP (unstable estimate, non-interpretable) reached significance.

**Table 5:** Multivariable logistic regression model for predictors of 28-day mortality.

Variable	Coefficient (B)	Standard error	Wald χ^2^	*p*-value	Odds ratio (OR)	95% CI Lower	95% CI Upper
KPC positivity	2.68	1.06	6.39	0.011	14.58	1.82	116.48
APACHE II ≥ 20	0.41	1.13	0.13	0.717	1.50	0.17	13.64
Mechanical power ≥18 J/min	−16.96	7654.69	<0.01	0.998	0.00	–	–
Constant	−1.99	0.67	8.22	0.004	–	–	–

The OR value for Mechanical Power ≥18 J/min was estimated as 0 (non-interpretable) due to model instability and the small number of events. The only independent predictor that remained statistically significant was KPC positivity (OR 14.6, 95% CI 1.8–116.5, *p* = 0.011). The model showed good overall fit: χ^2^ = 9.02, *p* = 0.029; Hosmer–Lemeshow *p* = 0.94; AUC = 0.801 (95% CI 0.62–0.92). Only KPC positivity remained an independent predictor of death. The OR for mechanical power ≥18 J/min was unstable (approximated to 0) due to sparse events. The model demonstrated good global fit (χ^2^ = 9.02, *p* = 0.029), calibration (Hosmer–Lemeshow *p* = 0.94).

## Discussion

This pilot study examined the association between mechanical power (MP) delivered during standardized ventilation prior to urgent bronchoscopy and major outcomes in critically ill patients. Contrary to our initial hypothesis, MP values were comparable between survivors and non-survivors. Instead, the detection of *Klebsiella pneumoniae* carbapenemase (KPC) in bronchoalveolar lavage emerged as the strongest single predictor of death, reinforcing multicenter findings on the prognostic dominance of carbapenem-resistant Enterobacterales in ventilated populations (14, 15).

Mechanical power integrates tidal volume, respiratory rate, driving pressure, and flow pattern into a single measure of ventilator energy load (16). Prior studies have demonstrated that higher sustained MP is associated with VILI and mortality, often identifying thresholds such as MP ≥ 18 J·min^−1^ (3). In our cohort, however, MP values were within a moderate range, and no independent association with mortality was found. This likely reflects the brief, standardized pre-bronchoscopy measurement period and lung-protective ventilator settings employed, which resulted in a relatively narrow range of MP values. Under these conditions, MP captures basal ventilatory status rather than dynamic changes under therapy.

Our findings align with accumulating evidence suggesting that cumulative duration of elevated MP exposure over days, rather than isolated pre-procedural measurements, drives VILI and mortality (3). Nevertheless, our standardized MP measurement protocol served important indirect functions: ensuring physiologic optimization before a high-risk intervention, creating reproducible baseline conditions that minimize procedural complications, establishing reference points for intra- and post-procedural ventilator adjustments, and generating high-quality data for research. Therefore, MP standardization should be viewed not primarily as a mortality prediction tool in this context, but rather as a component of comprehensive procedural safety optimization and quality assurance infrastructure.

In contrast to MP, KPC positivity remained the only independent predictor of 28-day mortality after adjustment, consistent with regional and international reports showing high mortality rates in KPC-related infections, particularly when adequate therapy is delayed (17). These results highlight the critical importance of rapid resistance profiling, such as multiplex PCR, for early risk stratification and targeted intervention (7, 17). We also observed that lower leukocyte counts were associated with mortality, consistent with sepsis-induced immunoparalysis as a poor prognostic marker (18).

Our findings have direct implications for clinical practice. First, in patients requiring urgent bronchoscopy, rapid microbiological diagnostics should be prioritized over repeated ventilatory assessments for mortality risk stratification. Implementing multiplex PCR panels that can detect KPC and other carbapenemase genes within 1–2 h may be more valuable than serial MP calculations for identifying high-risk patients requiring immediate escalation of care. Second, our results support a two-tier approach: (1) standardized ventilatory stabilization to ensure procedural safety, and (2) expedited molecular resistance profiling to guide prognostic discussions and therapeutic planning. Third, institutions should consider integrating rapid PCR results into clinical pathways and early warning systems, with KPC detection triggering automatic consultation protocols and enhanced infection control measures.

While our multivariable analysis appropriately separates the independent effects of MP and KPC, this statistical approach does not fully capture their complex interplay in real-world ICU environments. Standardized ventilatory protocols and infection control practices are complementary quality indicators that together reflect institutional commitment to evidence-based care. Both MP reduction and resistance control require sustained, coordinated effort across multiple disciplines and care processes. In settings where resistance prevalence is high and diagnostic capacity is limited, investment in rapid resistance profiling infrastructure may yield greater population-level mortality benefit than incremental refinements in ventilatory monitoring technology, though optimal ICU care requires parallel investment in both domains (19, 20).

An important consideration is that all data were collected in 2019, prior to the COVID-19 pandemic, which fundamentally transformed ICU practice patterns, patient case-mix, and healthcare systems globally. Post-COVID ICUs manage higher proportions of patients with prolonged mechanical ventilation, altered antimicrobial resistance patterns, and complex comorbidities (21). Additionally, structural and systemic barriers to optimal critical care—including gaps in vaccination coverage, (22, 23) antimicrobial stewardship program disruptions, and ICU capacity strain—profoundly influence outcomes and may modify the relationships we observed (24). While our core findings remain valid, prospective validation in contemporary ICU populations is essential before widespread implementation. (25)

This study has important limitations. First, the sample size was small (*n* = 30), limiting the statistical power to detect modest associations between mechanical power and outcomes and increasing risk of type II errors. The retrospective design is subject to potential misclassification and unmeasured confounding. Additionally, MP was measured only immediately prior to bronchoscopy, not continuously, which may underestimate cumulative impact.

Despite these limitations, the study has several strengths. The protocolized, standardized monitoring of ventilatory parameters and careful calibration of the pre-bronchoscopy steady-state period enhance the validity and reproducibility of physiologic data. All procedures were performed and monitored by experienced, critical care-trained operators, minimizing procedural variation and risk. The integration of advanced microbiological diagnostics, including multiplex PCR, enabled high-resolution pathogen and resistance profiling—a critical advance in modern ICU care.

Future studies should employ prospective designs with larger samples (≥150–200 patients), serial MP measurements throughout the ICU course, multicenter collaborations to enhance external validity, and integration of immunologic biomarker profiling alongside mechanical and microbiological variables. Implementation science studies evaluating real-world integration of rapid PCR-based resistance profiling into clinical workflows are also critically needed.

## Conclusion

In critically ill patients undergoing urgent fiberoptic bronchoscopy, standardized pre-procedural ventilatory conditions enabled reliable assessment of respiratory mechanics and mechanical power. Contrary to expectations, baseline MP did not independently predict short-term outcomes, whereas KPC-producing *Klebsiella pneumoniae* emerged as the predominant determinant of 28-day mortality. These findings underscore the prognostic dominance of microbiological resistance over transient physiologic parameters and highlight the need to integrate rapid resistance profiling with ventilatory monitoring to improve risk stratification and guide management in the ICU.

## Data Availability

The raw data supporting the conclusions of this article will be made available by the authors, without undue reservation.
